# Supplementary material for: Non-Insulin Antidiabetic Agents and Lung Cancer Risk in Drug-Naive Patients with Type 2 Diabetes Mellitus: A Nationwide Retrospective Cohort Study
Source: Cancers (Basel). 2024 Jun 28;16(13):2377. doi: 10.3390/cancers16132377 (PMC11240387; doi:10.3390/cancers16132377)
Supplement: Supplementary file 1 [file cancers-16-02377-s001.zip › cancers-3058218-supplementary.pdf]

Supplementary Table 2: Demographic Characteristics of the Cohorts After Propensity Score Ma

	%b GLP-1RA (+)/Insulin (-) (n=29,850)	Insulin (+)/GLP-1RA (-) (n=29,751)	SMDc	Metformin (+)/Insulin (-) (n=250,615)	Insulin (+)/Metformin (-) (n=249,277)	SMD	TZD(+)/Insulin(-) (n=24,861)	Insulin (+)/TZD(-)(n=24,744)
Age at Index Event, mean (SD), y	56.5(11.6)	56.5(12.5)	0.0121	60.5(13.3)	59.8(15.9)	0.0450	62.1(12.0)	62.0(12.4)
Sex								
Female	54.9	54.5	0.0077	48.9	48.8	0.0018	44.7	44.7
Male	39.4	39.8	0.0087	47.2	47.3	0.0016	53.4	53.3
Ethnicity								
Hispanic/LatinX	7.8	7.5	0.0098	9.5	9.6	0.0009	11.4	11.2
Not Hispanic/LatinX	68.6	69.0	0.0080	66.0	67.5	0.0321	63.8	63.9
Unknown	23.6	23.5	0.0026	24.5	23.0	0.0362	24.8	24.9
Race								
American Indian or Alaska Native	0.4	0.4	0.0018	0.4	0.4	0.0020	0.4	0.4
Asian	2.5	2.3	0.0134	4.3	3.9	0.0182	4.7	4.6
Black	13.2	13.6	0.0112	17.4	17.9	0.0148	12.9	13.1
Native Hawaiian or Other Pacific Islander	0.5	0.4	0.0120	0.6	0.6	0.0001	0.4	0.4
White	68.1	68.5	0.0079	61.9	62.0	0.0011	66.1	66.0
Unknown	13.1	12.7	0.0106	12.5	12.2	0.0069	12.5	12.6
Lifestyle Factors:								
Nicotine de	7.5	7.2	0.0118	10.4	10.1	0.0116	7.4	7.2
Personal h	6.1	5.5	0.0234	6.4	6.3	0.0045	4.9	4.8
Alcohol rel	1.3	1.1	0.0139	2.6	2.4	0.0120	1.5	1.5
Family History and Screening								
Family hist	0.3	0.2	0.0137	0.2	0.2	0.0043	0.1	0.1
Encounter	0.2	0.1	0.0304	0.1	0.1	0.0059	0.1	0.0
Personal h	0.1	0.1	0.0013	0.2	0.2	0.0018	0.1	0.1

<u>Pre-Existing Conditions</u>								
Asthma	11.1	10.8	0.0081	8.5	8.5	0.0008	7.1	6.9
Primary re:	0.0	0.0		0.0	0.0	0.0012	0.0	0.0
<u>Adverse Exposures:</u>								
Pneumoco	0.0	0.0	0.0000	0.0	0.0	0.0005	0.0	0.0
Contact wi	0.1	0.1	0.0013	0.1	0.1	0.0030	0.1	0.1
Contact wi	0.0	0.0	0.0258	0.0	0.0	0.0089	0.0	0.0
Personal h	0.4	0.3	0.0131	0.5	0.5	0.0041	0.4	0.4
<u>Hemoglobi</u>	7.7(1.8)	7.7(2.0)		7.4(1.7)	7.3(1.9)		7.7(1.7)	7.7(1.9)
>9 %	20.0	19.7	0.0064	11.4	11.4	0.0002	19.9	19.5
<9 %	53.9	53.6	0.0058	47.0	45.7	0.0277	55.7	55.3
No recorded A1c								
<u>BMI, mean</u>	36.3(6.6)	35.1(6.5)		32.9(6.8)	32.3(7.0)		33.0(6.6)	33.1(6.8)
0-18.4 kg/r	0.4	0.3	0.0165	0.4	0.4	0.0017	0.3	0.3
18.5-24.9 k	1.2	1.1	0.0103	3.0	3.0	0.0017	2.1	1.9
25-29.9 kg,	5.1	4.9	0.0119	7.4	7.4	0.0025	6.1	5.8
At least 30	18.8	17.7	0.0278	14.1	13.4	0.0193	12.5	12.2

Abbreviation: GLP-1RA, glucagon-like peptide 1 receptor agonist

aThe status of variables was based on the presence of related clinical codes anytime to 1 day before the index event. Other variables that were not shown

b A plus sign (+) indicates that a patient was prescribed the listed medication, while a minus sign (-) inc

c SMD less than 0.1, a threshold indicating group balance. SMD, standardized mean difference.

tching

SMD	SU(+)/Insulin(-) (n=81,620)	Insulin(+)/SU(-)(n=81,227')	SMD	DPP-4(+)/Insulin(-)(n=58,323)	Insulin (+)/DPP-4(-)(n=58,021)	SMD	AGI(+)/Insulin(-)(n=1,441)	Insulin (+)/AGI(-) (n=1423)	SMD
0.0116	62.7(13.2)	62.7(13.7)	0.0020	62.4(12.5)	62.3(12.9)	0.0063	61.7(14.7)	61.8(15)	0.0011
0.0006	45.1	45.3	0.0038	47.4	47.4	0.0002	53.4	53.0	0.0083
0.0012	49.9	49.6	0.0063	47.7	47.7	0.0002	43.5	43.5	0.0000
0.0061	9.2	9.1	0.0044	8.9	8.6	0.0077	7.6	8.0	0.0129
0.0018	70.9	71.0	0.0031	68.2	68.1	0.0024	69.9	67.8	0.0448
0.0024	19.9	19.9	0.0004	23.0	23.3	0.0079	22.5	24.2	0.0409
	0.4	0.4		0.4	0.4		0.4	0.4	
0.0019			0.0017			0.0013			0.0013
0.0074	4.2	4.0	0.0091	5.4	5.3	0.0061	6.6	6.4	0.0084
0.0052	16.5	16.8	0.0075	14.3	14.6	0.0082	14.1	14.9	0.0236
0.0033	0.5	0.4	0.0035	0.5	0.5	0.0054	0.7	0.7	0.0000
0.0013	64.1	64.3	0.0038	64.1	64.3	0.0038	64.8	64.6	0.0043
0.0034	11.6	11.4	0.0064	12.5	12.2	0.0065	10.9	11.2	0.0110
0.0074	9.1	8.7	0.0141	8.2	7.9	0.0101	8.0	7.3	0.0260
0.0024	6.7	6.6	0.0024	6.2	5.8	0.0151	7.8	7.4	0.0157
0.0003	1.9	1.7	0.0154	1.5	1.4	0.0050	2.1	1.6	0.0361
0.0128	0.2	0.2	0.0111	0.2	0.2	0.0185	0.7	0.7	0.0000
0.0177	0.1	0.1	0.0104	0.2	0.1	0.0277	0.0	0.0	
0.0046	0.2	0.1	0.0126	0.1	0.1	0.0052	0.7	0.7	0.0000

0.0086	7.9	7.5	0.0138	8.5	8.2	0.0113	11.4	11.5	0.0044
0.0283	0.0	0.0	0.0156	0.0	0.0	0.0000	0.0	0.7	0.1181
0.0283	0.0	0.0	0.0000	0.0	0.0	0.0000	0.0	0.0	
0.0108	0.1	0.1	0.0090	0.1	0.1	0.0115	0.0	0.0	
0.0000	0.0	0.0	0.0156	0.0	0.0	0.0185	0.0	0.0	
0.0087	0.5	0.5	0.0051	0.5	0.4	0.0126	0.7	0.7	0.0000
	7.7(1.8)	7.6(2.0)		7.8(1.7)	7.6(2.0)		7.3(1.7)	7.4(1.7)	
0.0099	18.1	17.2	0.0238	19.7	19.5	0.0062	17.3	17.8	0.0127
0.0089	53.0	52.7	0.0054	58.1	57.8	0.0067	60.8	60.7	0.0014
	32.8(6.7)	32.9(6.9)		32.8(6.6)	32.9(6.9)		31.8(7.0)	31.9(6.5)	
0.0141	0.4	0.3	0.0140	0.4	0.4	0.0110	0.9	0.7	0.0234
0.0145	2.9	2.7	0.0118	3.0	2.9	0.0057	4.7	3.9	0.0410
0.0161	7.4	6.9	0.0227	8.1	7.4	0.0258	11.1	10.6	0.0178
0.0091	14.4	13.7	0.0196	15.1	14.6	0.0157	15.8	15.9	0.0019

but propensity score matched include number of health care visits utilized before the index event.

dicates that they were not.

SGLT2 (+)/Insulin (-) (n=17,723)	Insulin (+)/SGLT2 (-) (n=17,694)	SMD
57.7(11.1)	57.5(11.8)	0.0152
40.9	40.9	0.0000
50.6	50.6	0.0008
9.1	8.9	0.0077
68.7	68.7	0.0009
22.2	22.4	0.0063
0.3	0.3	0.0020
5.1	4.9	0.0091
10.2	10.5	0.0112
0.8	0.7	0.0132
66.8	67.1	0.0055
14.4	14.2	0.0057
8.1	7.9	0.0091
6.4	5.9	0.0204
1.5	1.3	0.0122
0.3	0.2	0.0305
0.2	0.1	0.0284
0.1	0.1	0.0038

9.1	8.7	0.0147
0.1	0.0	0.0332
0.0	0.1	0.0332
0.1	0.1	0.0023
0.1	0.0	0.0332
0.4	0.3	0.0238
8.0(1.8)	7.8(2.1)	
24.4	24.0	0.0091
57.3	56.7	0.0117
34.4(6.6)	34.0(6.8)	
0.4	0.4	0.0081
1.9	1.8	0.0057
6.9	6.7	0.0098
18.1	17.5	0.0181
